# Methotrexate-Associated Lymphoproliferative Disorder in a Patient with Polymyalgia Rheumatica Presenting with Double Vision

**DOI:** 10.1080/01658107.2024.2365262

**Published:** 2024-06-17

**Authors:** Gabriele Berman, Rasoul Amel-Kashipaz, Prem Mahendra, Satheesh Ramalingam, Benjamin Rhodes, Susan Mollan, Ajay Patil

**Affiliations:** a Birmingham Neuro-Ophthalmology, University Hospitals Birmingham NHS Foundation Trust, Queen Elizabeth Hospital, Birmingham, UK; b Department of Pathology, University Hospitals Birmingham NHS Foundation Trust, Queen Elizabeth Hospital, Birmingham, UK; c Department of Haematology, University Hospitals Birmingham NHS Foundation Trust, Queen Elizabeth Hospital, Birmingham, UK; d Department of Neuroradiology, University Hospitals Birmingham NHS Foundation Trust, Queen Elizabeth Hospital, Birmingham, UK; e Department of Rheumatology, University Hospitals Birmingham NHS Foundation Trust, Queen Elizabeth Hospital, Birmingham, UK; f Translational Brain Science, Institute of Metabolism and Systems Research, College of Medical and Dental Sciences, University of Birmingham, Birmingham, UK

**Keywords:** Diffuse large B-cell lymphoma, FDG-PET/CT, giant cell arteritis, lymphoproliferative disorder, methotrexate, polymyalgia rheumatica

## Abstract

Methotrexate is a commonly employed folate antagonist used as a disease modifying antirheumatic drug. It is recommended by the European League Against Rheumatism Guidelines as an add-on therapy for the treatment of polymyalgia rheumatica. Lymphoproliferative disease developing during methotrexate treatment is recognised as methotrexate-associated lymphoproliferative disorder. We describe a patient with polymyalgia rheumatica on long-term methotrexate treatment presenting with double vision and systemic symptoms concerning for giant cell arteritis. Two months prior, she had noticed a mass of the right nasal dorsum. Neuroimaging showed several lesions of the nasal cavity and a clival lesion. Nasal cavity biopsy revealed diffuse large B-cell lymphoma, and FDG-PET/CT 3 weeks after methotrexate cessation showed significant interval disease regression, confirming the diagnosis of methotrexate-associated lymphoproliferative disorder. Follow-up FDG-PET/CT 4 months after methotrexate cessation showed complete radiological regression of lymphoproliferative lesions. The cumulative incidence of methotrexate-associated lymphoproliferative disorder in patients with rheumatoid arthritis treated with methotrexate has been reported to be up to 4.7% at 10 years in a retrospective study. Cessation of methotrexate resulted in spontaneous regression in 59% of patients. It is important to include methotrexate-associated lymphoproliferative disorder on the differential diagnosis for patients on long-term methotrexate treatment who present with neuro-ophthalmic symptoms and signs as tissue diagnosis prior to commencing steroid treatment is essential to secure the diagnosis and guide treatment.

## Introduction

Polymyalgia rheumatica (PMR) relies on a clinical diagnosis of predominantly proximal musculoskeletal pain and stiffness with raised inflammatory markers. Current treatment guidelines recommend minimum effective glucocorticoid treatment and early introduction of methotrexate in addition, especially in patients at high risk for relapse, prolonged therapy, or high risk of glucocorticoid adverse events.^1^ One in five patients with PMR will have or eventually develop giant cell arteritis (GCA).^2^ As GCA is a vasculitis affecting medium and large vessels, systemic symptoms are diverse and can include headache, fatigue, fever, myalgia, night sweats, loss of appetite, and weight loss.^3^ Important ophthalmic symptoms in GCA are transient monocular vision loss and transient or persistent double vision. Double vision can manifest due to oculomotor cranial nerve palsies or extraocular muscle ischaemia.^4^ It is important to maintain a high index of suspicion, as these symptoms often precede devastating and permanent vision loss.

## Case report

A 78-year-old Caucasian woman presented with initially intermittent binocular horizontal diplopia and decreased appetite. Six years prior, she had reported generalized muscle pain, and loss of appetite, elevated CRP (212 mg/L), and ESR (46 mm/Hr) were recorded. CT thorax, abdomen, and pelvis was unremarkable. She was diagnosed with PMR, and her symptoms resolved on 30 mg prednisone daily. As symptoms recurred on tapering of corticosteroids below 5 mg, methotrexate 15 mg weekly was commenced. Her PMR remained stable on prednisone 5 mg daily and methotrexate 10–20 mg weekly. Four years after PMR diagnosis, she developed a low-grade seronegative inflammatory arthritis, and hydroxychloroquine 200 mg was added daily.

At this admission, she presented with a four-day history of binocular horizontal diplopia and loss of appetite. The patient had noted a hard ‘’lump’’ at the right side of the nasal bridge 2 months previously. She denied transient vision loss, new headaches, or jaw claudication. On examination, her vision, colour vision, and visual fields were normal. Pupils were isocoric without relative afferent pupillary defect. She had a subtle left abduction deficit with slowing of abducting saccades. On dilated fundus examination, there was no optic disc swelling, retinal hemorrhages, or ischemia. A non-erythematous firm and adherent mass without pain on palpation of the right nasal dorsum was noted. Blood tests on admission included elevated CRP of 74 mg/L and ESR of 45 mm/Hr and normal leucocytes (6.2 × 10 × 9/L) and platelets (274 × 10 × 9/L). Methotrexate was discontinued due to concern for infection.

The main differential diagnoses to keep in mind in an immunosuppressed patient with new onset cranial nerve palsy are flare of known autoimmune disease, infection, and neoplasia, and further work-up including neuroimaging was pursued.

**Figure 1.**
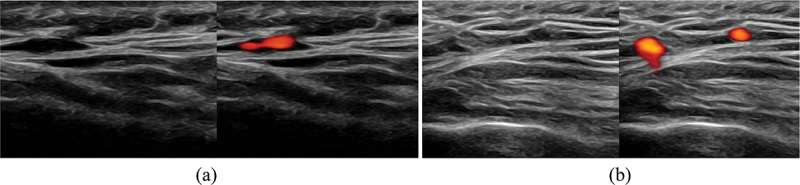
Normal ultrasound with colour Doppler of right superficial temporal artery without perivascular oedema or thickening; longitudinal (a) and cross-sectional (b) view.

**Figure 2.**
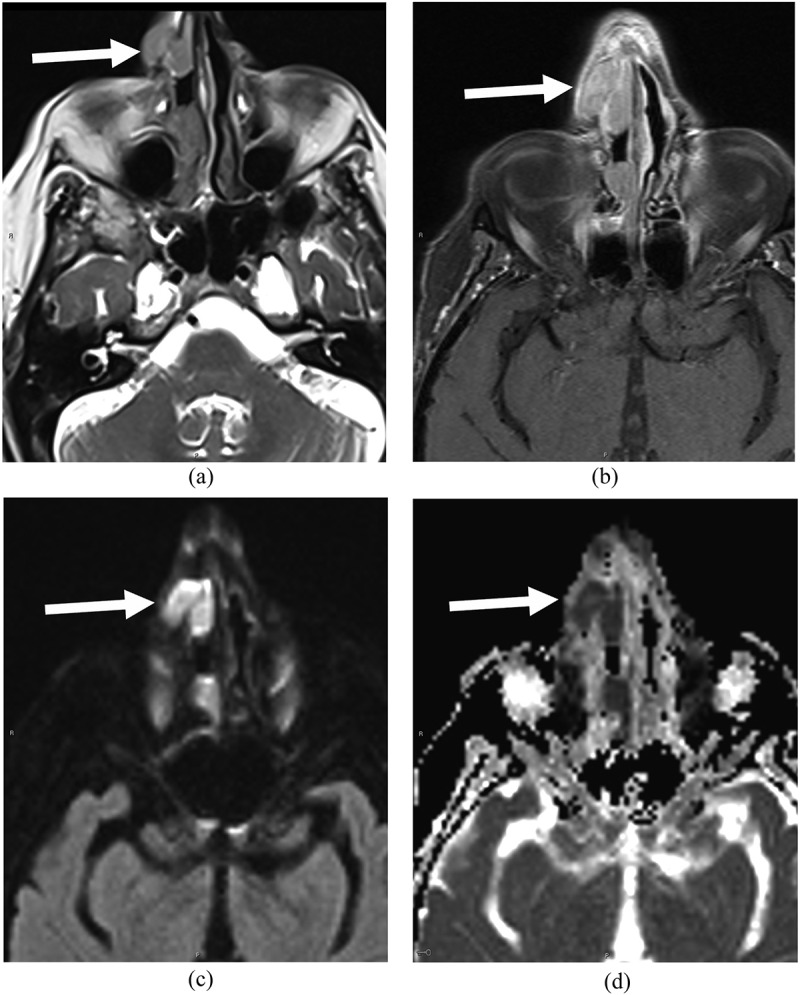
Axial MRI sinus showed soft tissue disease (arrows) in the right nasal cavity and right nasal alar region with low MRI T2 signal (a) and high T1 signal with contrast enhancement (b) and restricted diffusion on DWI/ADC (c, d).

**Figure 3.**
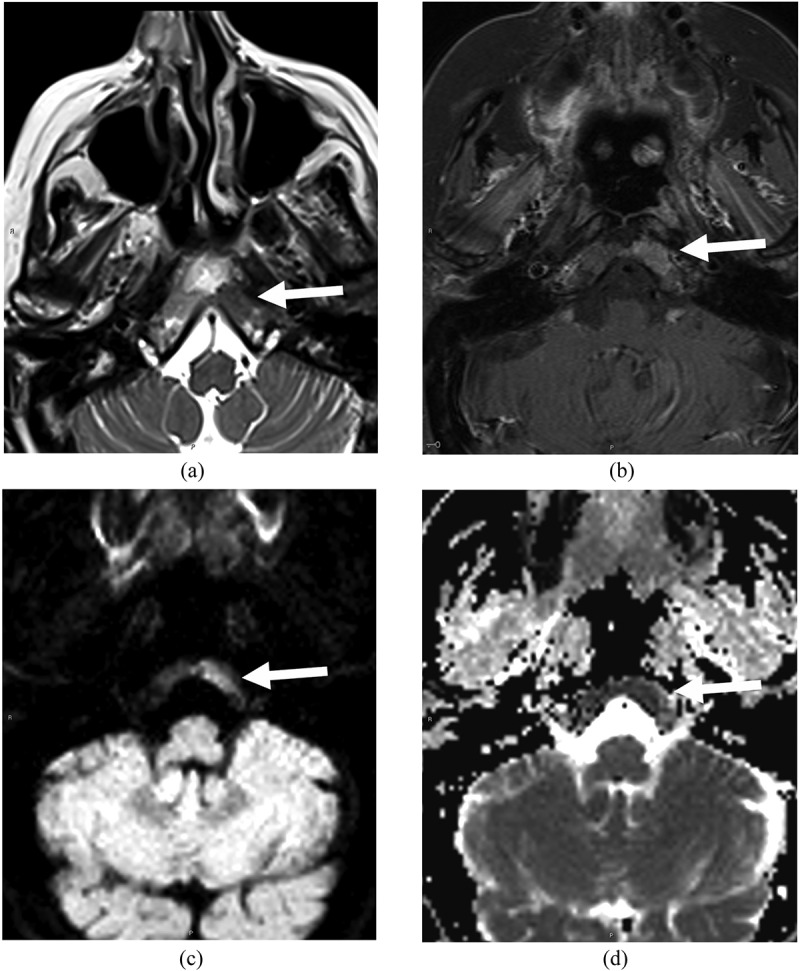
Axial MRI sinus showed disease infiltration in clivus (arrows) involving left Dorello’s canal with low MRI T2 signal (a) and high T1 signal with contrast enhancement (b) and restricted diffusion on DWI/ADC (c, d).

The clinical suspicion of GCA based on history and examination was low, and ultrasound of the common carotid arteries, axillary, and temporal arteries showed no perivascular edema or thickening (Figure 1). CT sinuses without contrast showed soft tissue disease in the nasal cavity and right nasal ala. MRI sinuses with contrast was obtained to better characterize the soft tissue disease and demonstrated three lesions of the nasal cavity and a clival lesion. Although the clival lesion did not radiologically exert mass effect on the brain stem, it was likely to cause localized compression of the abducens nerve on its ascend of the clivus prior to entering Dorello’s canal. All lesions displayed low MRI T2 signal and high T1 signal with contrast enhancement and restricted diffusion (Figures 2 and 3). Tissue diagnosis was essential to characterize the disease process, and as all lesions displayed similar image characteristics, endoscopic endonasal biopsy of the accessible lesion of the nasal cavity was planned.

**Figure 4.**
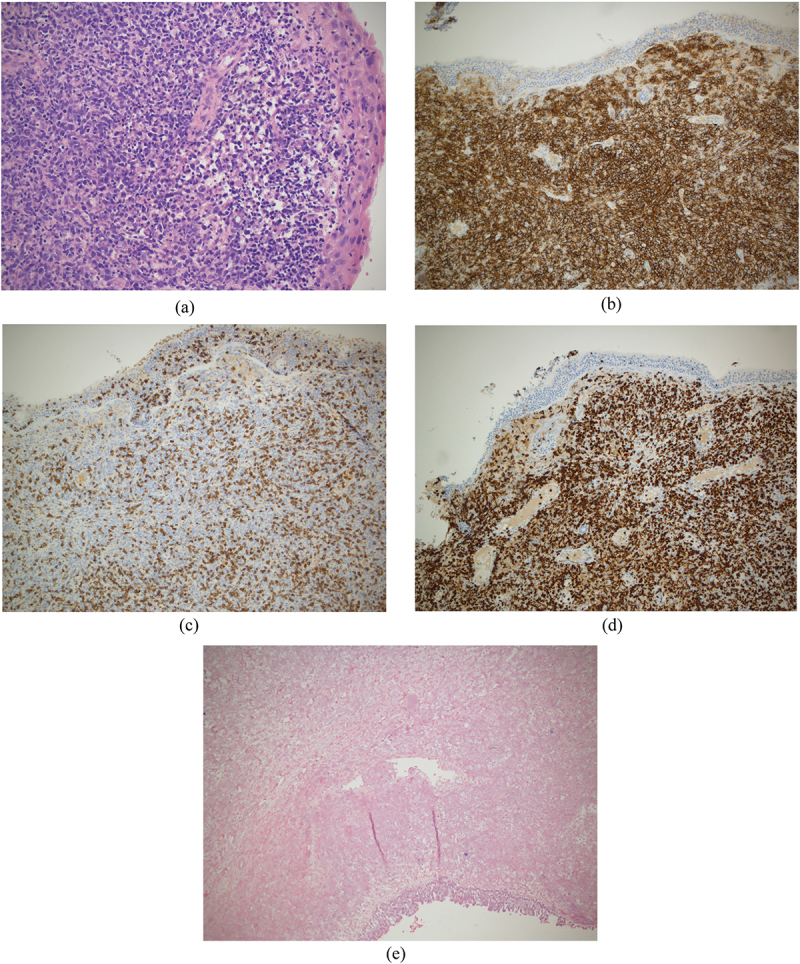
Right nasal cavity biopsy.

The endoscopic right nasal cavity biopsy showed squamous lined mucosa with diffuse sheets of atypical lymphocytes with enlarged nuclei, increased mitoses, and apoptotic particles. The lymphocytes were positive for CD20, CD79a, MUM-1, and BCL-2 but negative for CD30, CD10, BCL 6, cyclin D1, and CD65. The proliferation index ki-67 was high (70%). The lymphocytes were negative for in situ hybridization for Epstein-Barr virus (EBV)-encoded RNA (Figure 4a–e). The histopathological diagnosis was Epstein-Barr virus-negative diffuse large B-cell lymphoma of non-germinal centre phenotype. The biopsy was composed entirely of lymphoma without normal background tissue to evaluate for vasculitis. EBV serology was positive (EBV PCR 2429 IU/ml). The patient continued on prednisolone 5 mg daily, without restarting methotrexate treatment.

(a) 200× H&E. Large atypical lymphocytes with increased mitoses and apoptotic particles.

(b) 100× CD20. Large-sized lymphoma cells positive for CD20 (B-cell marker).

(c) 100× CD3. Large-sized lymphoma cells negative for CD3 (T-cell marker).

(d) 100× ki-67. Lymphoma cells show high ki-67 proliferation index (70%).

(e) 100× EBV-ISH. In situ hybridization negative for EBV-encoded RNA.

**Figure 5.**
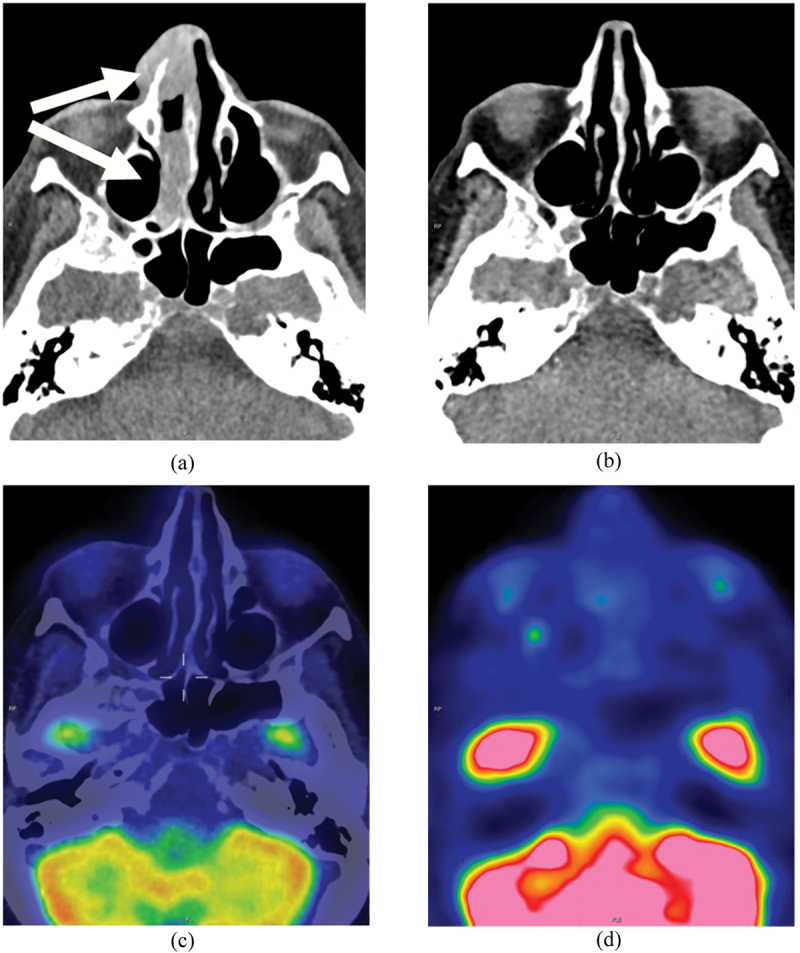
CT sinus at presentation showed soft tissue disease in the nasal cavity and right nasal ala (arrows) (a). CT sinus 3 weeks after methotrexate cessation showed regression of soft tissue disease in right nasal cavity and right nasal ala (b). FDG-PET/CT performed 3 weeks after methotrexate cessation showed disease regression (c, d).

**Figure 6.**
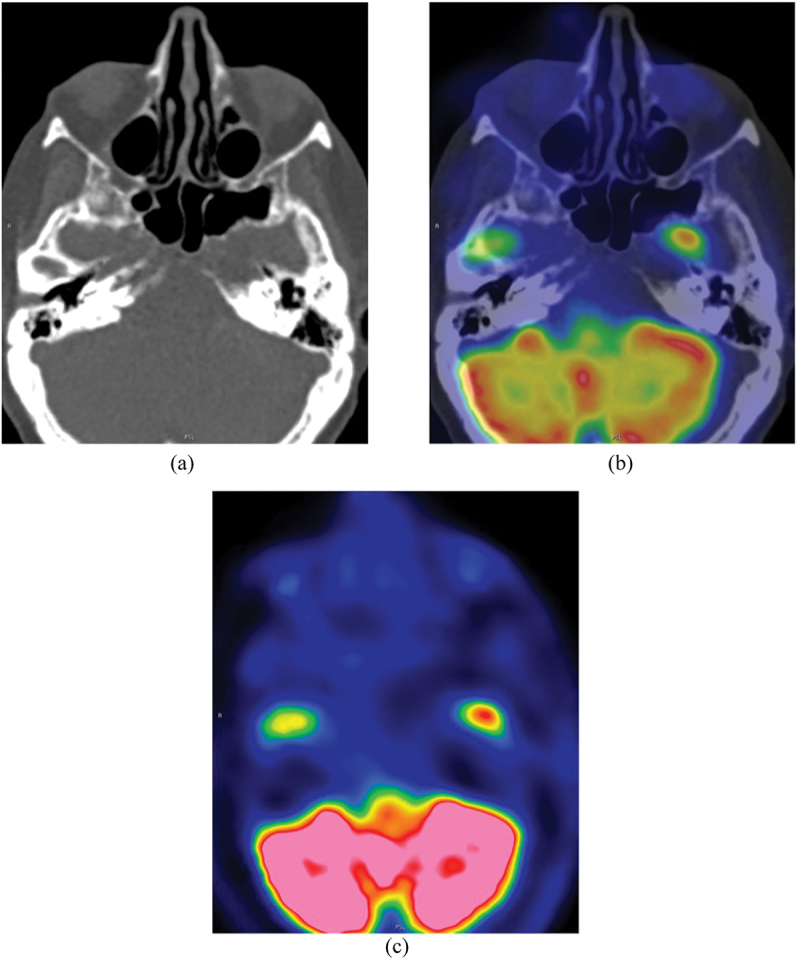
CT (a) and FDG-PET/CT (b, c) 4 months after methotrexate cessation showed continued disease regression.

Two and half weeks after methotrexate cessation, the right nasal dorsum mass was no longer clinically visible. Three weeks after methotrexate cessation, 18-fluorodeoxyglucose positron emission tomography (FDG-PET)/CT imaging vertex to mid-thigh showed marked interval improvement in the CT appearance with disease regression and minimal residual soft tissue thickening at the right philtrum (Figure 5). There was no PET/CT evidence for vasculitis of large and medium-sized vessels. A diagnosis of methotrexate-associated lymphoproliferative disorder was made. There was no measurable residual disease on PET/CT, and the patient was continued on low dose prednisolone. Chemotherapy was not commenced as per hematological guidelines for cases where methotrexate withdrawal results in substantial tumor regression clinically and radiologically. Follow-up FDG-PET/CT 4 months after initial presentation showed continued complete radiological regression/remission (Figure 6).

## Discussion

Immunosuppressive therapy is being used increasingly more and at an earlier stage in inflammatory and autoimmune diseases with the overarching principles of choosing treatment based on disease severity and activity and the aim of achieving and maintaining remission with the minimal effective dose of medication.^1,5^ Lymphoproliferative disorders that arise during immunosuppressive treatment are classified as iatrogenic immunodeficiency-associated lymphoproliferative disorders.^6^ Lymphoproliferative disease developing during methotrexate treatment is recognized as methotrexate-associated lymphoproliferative disorder (MTX-LPD). MTX-LPD has been reported predominantly in patients with rheumatoid arthritis (RA), likely due the prevalence of the disease and methotrexate being the recommended first line treatment.^7^ Methotrexate is a folate antagonist that inhibits folate-dependent enzymes as well as synthesis of purine and pyrimidine required for DNA and RNA production.^8^ The ambition to inhibit the inflammatory response in autoimmune diseases with methotrexate comes with the risk of lymphocytopenia.^9^ Association between methotrexate treatment and lymphoma was first reported in 1991 in rheumatoid arthritis patients treated with methotrexate.^10,11^ Later the term MTX-LPD was introduced to describe the occurrence of lymphoproliferative disorders in patients treated with methotrexate. Efforts have been made to further characterize the features and possible biomarkers of MTX-LPD. A retrospective study found 26/986 (2.6%) RA patients treated with methotrexate to develop LPD during a median follow-up of 78.3 months.^12^ A retrospective study comparing FDG-PET/CT biomarkers in biopsy-proven LPD in RA found metabolic tumor volume and total lesion glycolysis to be significantly lower among patients with spontaneous regression compared to patients requiring chemotherapy.^13^ The prevalence of EBV positivity among patients with RA and LPD was 54% in a metanalysis.^14^ Cessation of methotrexate resulted in spontaneous regression in 59% (28/47) of RA patients, and EBV positivity and non-DLBCL histology was significantly associated with spontaneous regression.^15^ Al-though the mechanism is not fully elucidated, it is thought that excessive inhibition of TH1-cells, effector memory CD8+T-cells, and EBV-specific CD8+ T-cells by methotrexate at the time of LPD development and their restoration after methotrexate cessation are specific features of the development and regression of LPD.^16^

MTX-LPD presenting with neuro-ophthalmic or neurological symptoms remains a rare entity requiring high index of suspicion. A recent systematic review reported a total of 13 cases of biopsy-verified CNS MTX-LPD in patients with RA, neurosarcoidosis, and dermatomyositis treated with MTX.^17^

In the case presented, the symptoms and elevated inflammatory markers were concerning for GCA in the setting of a known diagnosis of PMR. Initial differential diagnoses included inflammatory and infectious diseases. While awaiting investigations, glucocorticoids are obligatory treatment for suspected GCA but caution is required as it can be potentially devastating in infection. It is also important to consider the masking of lymphoproliferative disorders with glucocorticoid treatment when further radiological or histological tests are imminent. A full systems review is required in each patient presenting with neuro-ophthalmic disease, as in this case the nasal lesion warranted investigation and secured the diagnosis. Right nasal cavity biopsy showed EBV-negative diffuse large B-cell lymphoma of non-germinal centre phenotype. Discontinuation of methotrexate led to clinical improvement within 2.5 weeks and continued complete radiological regression/remission was documented by FDG-PET/CT four months after initial presentation.

There is only one prior case report describing lymphoproliferative disorder in a patient with PMR treated with methotrexate.^17^ There is changing opinion that PMR and GCA are actually one underlying condition, and the clinical phenotype is a reflection of the location of affected arterial tree.^18^ Glucocorticoids remain a cornerstone of treatment, but second line therapies are often required. There is class I evidence for the directed use of tocilizumab in new onset and relapsing GCA. In some healthcare settings, due to access, other non-biologic immunosuppression agents are considered before tocilizumab such as methotrexate and leflunomide as adjunctive therapy to avoid the side effects of glucocorticoids.^19^ It could be anticipated that there may be more patients with PMR-GCA spectrum disorder that may be affected by MTX-LPD, in view of the previously reported prevalence and increasing prevalence of the condition.^20,21,22^

## Conclusion

The final diagnosis was methotrexate-associated diffuse large B-cell lymphoma of right nasal cavity and clivus with spontaneous regression after methotrexate cessation. MTX-LPD should be considered in patients on long-term methotrexate presenting with new onset neurological symptoms. The diagnosis requires awareness of the disease entity and appropriate clinical index of suspicion to secure histological verification prior to steroid treatment. Methotrexate cessation is the initial first line treatment for MTX-LPD.

## References

[1] Dejaco C, Singh YP, Perel P, Hutchings A, Camellino D, Mackie S, et al. Recommendations for the management of polymyalgia rheumatica: a European league against rheumatism/American college of rheumatology collaborative initiative. *Ann Rheum Dis*. October, 2015;74(10):1799–1807. doi: 10.1136/annrheumdis-2015-207492. 26359488

[2] Salvarani C, Cantini F, Hunder GG. Polymyalgia rheumatica and giant-cell arteritis. *Lancet*. 2008;372(9634):234–245. doi: 10.1016/S0140-6736(08)61077-6. 18640460

[3] Lyons HS, Quick V, Sinclair AJ, Nagaraju S, Mollan SP. A new era for giant cell arteritis. *Eye*. June, 2020;34(6):1013–1026. doi: 10.1038/s41433-019-0608-7. 31582795 PMC7253415

[4] Bilton EJ, Mollan SP. Giant cell arteritis: reviewing the advancing diagnostics and management. *Eye*. August, 2023;37(12):2365–2373. doi: 10.1038/s41433-023-02433-y. 36788362 PMC9927059

[5] Dejaco C, Kerschbaumer A, Aletaha D, Bond M, Hysa E, Camellino D, et al. Treat-to-target recommendations in giant cell arteritis and polymyalgia rheumatica. *Ann Rheum Dis*. January, 2024;83(1):48–57. doi: 10.1136/ard-2022-223429. 36828585 PMC10803996

[6] Swerdlow SH, Campo E, Pileri SA, Harris NL, Stein H, Siebert R, et al. The 2016 revision of the World Health Organization classification of lymphoid neoplasms. *Blood*. 2016;127(20):2375–2390. doi: 10.1182/blood-2016-01-643569. 26980727 PMC4874220

[7] Smolen JS, Landewé RBM, Bergstra SA, Kerschbaumer A, Sepriano A, Aletaha D, et al. EULAR recommendations for the management of rheumatoid arthritis with synthetic and biological disease-modifying antirheumatic drugs: 2022 update. *Ann Rheum Dis*. January, 2023;82(1):3–18. doi: 10.1136/ard-2022-223356. 36357155

[8] Bedoui Y, Guillot X, Sélambarom J, Guiraud P, Giry C, Jaffar-Bandjee MC, et al. Methotrexate an old drug with new tricks. *Int J Mol Sci*. 2019;20(20):5023. doi: 10.3390/ijms20205023. 31658782 PMC6834162

[9] Buttgereit F, Dejaco C, Matteson EL, Dasgupta B. Polymyalgia rheumatica and giant cell arteritis: a systematic review. *JAMA*. 2016;315(22):2442. doi: 10.1001/jama.2016.5444. 27299619

[10] Shiroky JB, Frost A, Skelton JD. Complications of immunosuppression associated with weekly low dose methotrexate. *J Rheumatol*. August, 1991;18(8):1172–1175. 1941818

[11] Ellman MH, Hurwitz H, Thomas C. Lymphoma developing in a patient with rheumatoid arthritis taking low dose weekly methotrexate. *J Rheumatol*. November, 1991;18(11):1741–1743. 1787499

[12] Tanaka K, Ichikawa A, Umezawa N, Yamamoto K, Yoshifuji K, Okada K, et al. Lymphoproliferative disorder risk after methotrexate treatment for rheumatoid arthritis. *Cancer Sci*. September, 2023;114(9):3719–3727. doi: 10.1111/cas.15894. 37365854 PMC10475769

[13] Kameda T, Nakashima S, Mitamura K, Yamamoto Y, Norikane T, Shimada H, et al. FDG-PET/CT imaging parameters for predicting spontaneous regression of methotrexate-associated lymphoproliferative disorder in patients with rheumatoid arthritis. *Sci Rep*. 2022;12(1):15367. doi: 10.1038/s41598-022-19727-y. 36100660 PMC9470546

[14] Banko A, Miljanovic D, Lazarevic I, Jeremic I, Despotovic A, Grk M, et al. New evidence of significant association between EBV presence and lymphoproliferative disorders susceptibility in patients with rheumatoid arthritis: a systematic review with meta-analysis. 2022;*Viruses*. 14(1):115. doi: 10.3390/v14010115. 35062319 PMC8781518

[15] Ichikawa A, Arakawa F, Kiyasu J, Sato K, Miyoshi H, Niino D, et al. Methotrexate/iatrogenic lymphoproliferative disorders in rheumatoid arthritis: histology, E pstein– B arr virus, and clonality are important predictors of disease progression and regression. *Eur J Haematol*. July, 2013;91(1):20–28. doi: 10.1111/ejh.12116. 23560463

[16] Saito S, Suzuki K, Yoshimoto K, Kaneko Y, Yamaoka K, Shimizu T, et al. Restoration of decreased t helper 1 and CD8+ T cell subsets is associated with regression of lymphoproliferative disorders developed during methotrexate treatment. *Front Immunol*. 2018;9:621. doi: 10.3389/fimmu.2018.00621. 29670617 PMC5893782

[17] In A, Stopa BM, Cuoco JA, Stump MS, Apfel LS, Rogers CM. Central nervous system lymphoproliferative disorder secondary to methotrexate: a systematic literature review and case illustration. *World Neurosurg*. November, 2023;179:118–126. doi: 10.1016/j.wneu.2023.08.018. 37574195

[18] Ishibuchi H, Motegi SI, Yamanaka M, Amano H, Ishikawa O. Methotrexate-associated lymphoproliferative disorder: sequential development of angioimmunoblastic T-cell lymphoma-like lymphoproliferation in the lymph nodes and diffuse large B-cell lymphoma in the skin in the same patient. *Eur J Dermatol*. July, 2015;25(4):361–362. doi: 10.1684/ejd.2015.2582. 26105783

[19] Bosch P, Espigol-Frigolé G, Cid MC, Mollan SP, Schmidt WA. Cranial involvement in giant cell arteritis. *Lancet Rheumatol*. 2024;6:e384–e396. doi: 10.1016/S2665-9913(24)00024-9. 38574747

[20] Mackie SL, Dejaco C, Appenzeller S, Camellino D, Duftner C, Gonzalez-Chiappe S, et al. British society for rheumatology guideline on diagnosis and treatment of giant cell arteritis: executive summary. *Rheumatology*. 2020;59(3):487–494. doi: 10.1093/rheumatology/kez664. 31970410

[21] Mollan SP, Begaj I, Mackie S, O’Sullivan EP, Denniston AK. Increase in admissions related to giant cell arteritis and polymyalgia rheumatica in the UK, 2002–13, without a decrease in associated sight loss: potential implications for service provision. *Rheumatology*. 2015;54(2):375–377. doi: 10.1093/rheumatology/keu433. 25413943

[22] Li KJ, Semenov D, Turk M, Pope J. A meta-analysis of the epidemiology of giant cell arteritis across time and space. *Arthritis Res Ther*. December, 2021;23(1):82. doi: 10.1186/s13075-021-02450-w. 33706808 PMC7948334

